# Supplementary material for: Genome-wide association study of delay discounting identifies 11 loci and reveals transdiagnostic associations across mental and physical health
Source: Mol Psychiatry. 2025 Nov 25;31(4):2081–93. doi: 10.1038/s41380-025-03356-8 (PMC12999501; doi:10.1038/s41380-025-03356-8)
Supplement: Supplementary file 3 — README Top Independent SNPs [file 41380_2025_3356_MOESM3_ESM.docx]

**STRATEGY FOR SELECTING THE TOP 10,000 SNPS FOR PUBLIC RELEASE**

To select the most informative SNPs for public release, we clumped the top 10,000 SNPs with the lowest (most significant) p-values, using the approach described in Wray et al (2018), using the following clumping criteria:

- p1 and p2 = 1
- window = 500kb
- LD Rsq > 0.1

**Columns**

snpid – rsID

chr – chromosome

bp – position

A1 – effect allele

A2 – other allele

EAF – effect allele frequency

pvalue – p-value

effect – effect

se – standard error

zscore – z-score

**Data Availability**

As described in the Methods, full GWAS summary statistics for the 23andMe dataset will be made available to qualified researchers under an agreement with 23andMe that protects the privacy of the 23andMe participants. Please visit <https://research.23andme.com/collaborate/#dataset-access/> for more information and to apply to access the data.

**Acknowledgements**

This strategy was adapted from the PGC Major Depression 2 GWAS results public data release:

PGC-MDD2, Wray, Ripke, Mattheisen, Trzaskowski et al. (2018) Genome-wide association analyses identify 44 risk variants and refine the genetic architecture of major depression. Nature Genetics, 2018. PMID: [29700475](https://pubmed.ncbi.nlm.nih.gov/29700475/)
